# Its Uses and Technique

**Published:** 1921-01-01

**Authors:** 


### SPINAL ANÆSTHESIA.

### Its Uses and Technique.

For a few years after its discovery this type of anæsthesia enjoyed great popularity, and although the course of time has not justified the somewhat extravagant claims made for it, yet at the present time it has a definite though limited sphere of usefulness. The essential factor in the production of this anæsthesia is the injection of a solution of stovaine and glucose into the cerebro-spinal fluid at the average level of the third spinous process of the lumbar vertebræ. Then ensues an anæsthesia which extends upwards to the costal margins, and permits of operation below that level.

### Technique.

The patient should be prepared for an operation in the recognised routine fashion : that is, a purge on the night preceding operation, a good night's sleep, no solid food for at least four hours prior to operation, and a pre-anæsthetic dose of atropine and morphia. The dosage of the pre-anæsthetic injection should vary with the constitution of the patient. As regards atropine, $\frac{1}{100}$ of a grain may be considered a safe and efficient quantity for almost all persons above the age of twelve years, but the morphia dose should range between $\frac{1}{6}$ of a grain for a delicate woman to as much as $\frac{1}{3}$ of a grain for a robust, muscular man; the object of the soporific being to induce a tranquil state of mind and thus render the actual spinal injection of small import as far as the patient is concerned. Some anæsthetists favour the pre-anæsthetic injection of hyoscine hydrobromide, or so-called "Twilight sleep," but it seems likely that this drug tends to intensify the toxic symptoms tending to occur from time to time with the use of stovaine.

The patient is best brought into the operating-theatre for the actual spinal injection, and is placed upon the rigid operating-table. He is then put into the left lateral position and is instructed to bring the knees as near the chin as possible; this will produce a convex arching of the lumbar spine, separating the laminæ of the vertebræ and rendering the subsequent injection of the anæsthetic an easy task.

We cannot lay too much emphasis on these simple directions, because it is upon them that the whole success of the needle injection depends; for instance, one has observed the inexperienced anæsthetist attempting an injection in bed, a proceeding which needs much more luck than judgment to be successful, because the mattress of the bed is mobile and the weight of the patient will produce a scoliosis displacing the spine and making the injection a matter of great difficulty.

Once a correct posture is attained, the skin must be cleansed with ether, and afterwards painted with iodine, which should extend outwards in a thin line to the height of the iliac crest. The lumbar spinous process on a level with this line should now be sought—a matter of some difficulty in an adipose subject—and the needle introduced in the inter-spinous space immediably below the spine above. The patient should be warned before the needle prick is made, but if the needle be a sharp one and the skin pierced quickly but little inconvenience will be felt, for the tactile sensibility and discrimination is physiologically low in this region. The actual insertion of the needle needs a special word of description The stance of the anæsthetist should be as firm as possible, and so it is best to rest on one knee or else to stand with the legs far apart; the thumb of the left hand is then held firmly on the required spine and the needle held lightly in the palm of the right hand; just prior to injection the operator should be quite sure that his needle is almost at right angles to the spine, but with the point directed slightly upwards. The skin is then pierced and soon the resistance of the ligamentary tissues are felt and traversed into the spinal canal; the stilette of the needle is now withdrawn, and about 4 c.c. of the cerebro-spinal fluid allowed to escape. The nozzle of an air-empty sterilised syringe containing 1 c.c. of stovaine and glucose is now inserted into the open end of the needle. The pressure of the cerebro-spinal fluid will then gently push back the piston so that it mixes with the stovaine. The plunger is then lightly pushed down, this causing "mixed" fluid to pass back into the spinal canal, and the needle quickly withdrawn. A piece of adhesive strapping is then applied to the skin puncture, the patient immediately turned on his back; the head is then raised on two pillows, and the legs elevated so that the thighs lie at right angles to the trunk. The patient will almost immediately feel a not unpleasant sense of warmth and weight in the extremities, which is succeeded by a sensation of "pins and needles" quickly lapsing into paraplegia.

### A Personal Experience of Spinal Analgesia.

Operation for the radical cure of right inguinal hernia : Stovaine was injected between second and third lumbar spines, with patient lying on right side of table with his knees drawn up and lumbar region arched. Slight prick of needle in the skin felt. Patient was afterwards surprised to learn that the pin-prick had been the injection. After a minute or two a warm glow spread down the right leg, accompanied by tingling and feeling as if the leg were swollen. Five minutes after injection pin-prick in third lumbar region was felt only as a pressure. About ten minutes after injection, patient having been turned on his back, the operation was commenced. Any slight sense of operation seemed to refer to the left side instead of the right. About forty minutes after injection sensation began to return; about two hours after injection all signs of the action of stovaine seemed to have disappeared. As sensation returned pain was felt in the wound; it lasted for about six hours and then passed off.

### The Choice of Intra-spinal Drugs.

The ideal solution for anæsthesia should be of such strength as to be of the same osmotic tension as the blood serum. It should produce neither shrinking nor swelling of the blood or tissue cells by osmosis. Although many solutions have been experimented with, we consider a five per cent. solution of stovaine mixed with five per cent. of glucose to be the safest and most reliable in action; enough solution to comprise 0.05 grammes being injected as an average dose.

As an alternative we suggest the stovaine-dextrin solution of Tyrrel Gray, which contains stovaine three per cent. with dextrin and suprarenin, and has been used at the Great Ormond Street Hospital for Sick Children. The diminished toxicity of stovaine

**Spinal Anæsthesia—(*continued*).**

is due to the fact that it is very slowly diffusible; and this property is accelerated by administering it with diffusible fluids. Dextrin is a suitable addition, it being readily soluble, and innocuous to the tissues; furthermore, it appears to play a considerable part in controlling vomiting, and also delays absorption, so that no cases of stovaine poisoning were observed by its use. Normal saline is also used in strength isotonic with cerebro-spinal fluid, for it has been shown that though the diffusibility of dextrin-stovaine solution is above that of cerebro-spinal fluid when estimated in relation to water, yet when introduced into cerebro-spinal fluid, changes take place which make its diffusibility considerably less than laboratory experiments indicate. Pain, even after most severe operations, so slight that it was rarely, if ever, necessary to give hypnotics. Food, except where contra-indicated, can be given immediately after the operation, if wanted.

At the International Congress of Surgery in Brussels in 1908, Professor Jonnesco, of Bucharest, described a method of producing high analgesia by means of which he has been able to operate on every part of the body without interference with the respiratory or cardiac centres. In order to accomplish this Jonnesco used a combined solution of neutral sulphate of strychnine and stovaine, the former drug being used for the purpose of stimulating the respiratory and cardiac nerves, which it is enabled to do owing to its action being more rapid than that of stovaine.

The site of vertebral puncture in this case is between the first and second thoracic spines, the head and shoulders are then gently lowered, and analgesia becomes complete within two or three minutes for head operations, and five to seven minutes for thoracic.

This method must, however, for the present be considered as *sub judice*, for, although Jonnesco reported 156 high analgesias without untoward result, experiments in this country have been associated with several instances of respiratory failure.

### Advantages of Spinal Analgesia.

In operations such as complete excision of the rectum, the "Wertheim" removal of the uterus, and prostatectomy, the patient can very advantageously be spared much shock by this method.

According to Professor Crile's theory of anociassociation, the painful afferent symptoms are in great measure "blocked" by the zone of anæsthesia, and clinical experience certainly agrees with this supposition. Again, the very complete relaxation of the abdominal musculature, and the absence of unequal or violent respiratory motions, make the task of the surgeon—always a difficult one in these cases—more easy, and thus the operation takes less time to be completed. In cases where a general anæsthetic is definitely contra-indicated, as in glycosuria, chronic bronchitis, albuminuria and grave uræmic or cardiac states, this method is of unique usefulness.

The question of complete muscular relaxation is one which is exercising the minds of anæsthetists a great deal at the present time, and the most modern school of thought has veered towards the dictum that a general anæsthetic should never be "pushed" so far as to secure such a result: in which case the accessory use of spinal analgesia promises to be more extensively used than at the present time.

